# Primary Eye Care in Eastern Nepal

**DOI:** 10.1080/09286586.2019.1702217

**Published:** 2019-12-16

**Authors:** Helen Burn, Lila Puri, Abhishek Roshan, Sanjay K. Singh, Matthew J. Burton

**Affiliations:** a International Centre for Eye Health, Department of Clinical Research, Faculty of Infectious and Tropical Diseases, London School of Hygiene & Tropical Medicine, London, UK; b Sagarmatha Choudhary Eye Hospital, Lahan, Nepal; c Biratnagar Eye Hospital, Biratnagar, Nepal

**Keywords:** Primary eye care, eye health systems, Nepal, primary eye health workers, human resources for eye health

## Abstract

**Purpose**: Vision 2020 and the Global Action Plan 2013–2019 prioritise primary eye care (PEC) as an important component of reducing avoidable blindness. Studies in sub-Saharan Africa have demonstrated that current PEC provision is poor. There has been no evaluation of the current practice of PEC among primary health care workers (PHCWs) in Nepal.

**Methods**: A mixed methods descriptive cross-sectional study with semi-structured interviews and focus group discussions (FGDs) was carried out in Eastern Nepal. Government employed PHCWs working at health posts in three districts were invited to take part in a PEC knowledge and skills assessment. Each health post was assessed for ophthalmic equipment and medications. Three focus group discussions and eight semi-structured interviews were carried out with community ophthalmic assistants, PHCWs and a district health manager.

**Results**: 107 PHCWs in 35 health posts took part in the quantitative study. Only 8.4% had received eye care training. 27.1% PHCWs could diagnose a corneal ulcer, 83.2% conjunctivitis, 75.7% cataract and 54.2% ophthalmia neonatorum. Only 14.0% could measure visual acuity, and 5.7% of HPs had a vision chart. Ophthalmic assistants described their concern for the low level of PEC at health posts. PHCWs were keen to receive training and highlighted the need for greater government support in the provision of eye care services.

**Conclusion**: PEC knowledge and skills among PHCWs in eastern Nepal is inadequate to provide quality PEC services. There is a pressing need for PEC training in the region, provision of ophthalmic equipment and greater government support for eye care.

## Introduction

Worldwide there are 36.0 million blind and 216.6 million visually impaired.^1^ Of these, the majority of cases are preventable or treatable.^2^ With most eye care being delivered through secondary and tertiary levels of the health system, initial access to services remains a barrier to addressing visual impairment. A key approach to improving access is the integration of eye care into primary health care to provide treatment and referral at the lowest population level to identify those in need of eye health services.

The key components of PEC have been consistently stated as “promotive, preventative, curative and rehabilitative”.^3–7^ Vision 2020 placed PEC as an integral part of achieving their goals referring to the role of PEC as “promotion of eye health and/or the provision of basic preventive and curative treatment for common eye disorders”.^8^ Following this the Global Action Plan 2013–2019 described primary eye health workers as a key component of human resources for eye health.^9^

There is limited research examining the population need for PEC. A study in Rwanda found that one third of the population had the potential to benefit from PEC including refractive services, referral to secondary eye care and basic primary treatment.^10^ While there is belief that delivering eye care at the most basic community level would help to reduce avoidable blindness by improving access to eye care, there is limited evidence evaluating whether it is a successful approach.^11^ Across many low-income countries there are a wide range of different PEC models used, with little evaluation of whether interventions have been effective.

The PEC knowledge and skills of primary healthcare workers (PHCWs) in Tanzania, Kenya and Malawi was shown to be low.^12^ Similar results were found in Ethiopia and Nigeria.^13,14^ Other studies have evaluated the effectiveness and long-term impact of PEC training. In Tanzania an evaluation of PEC knowledge and skills pre and post PEC training showed an improvement in knowledge in the short-term, but without long term sustainability.^15^ The impact of PEC training on patient referrals to eye facilities was assessed in Rwanda and showed an increase in eye-care visits to health centres and referrals for cataract surgery for the first 6 months, however this initial increase in service use was not maintained over the following 2 years.^16^

**Figure 1.**
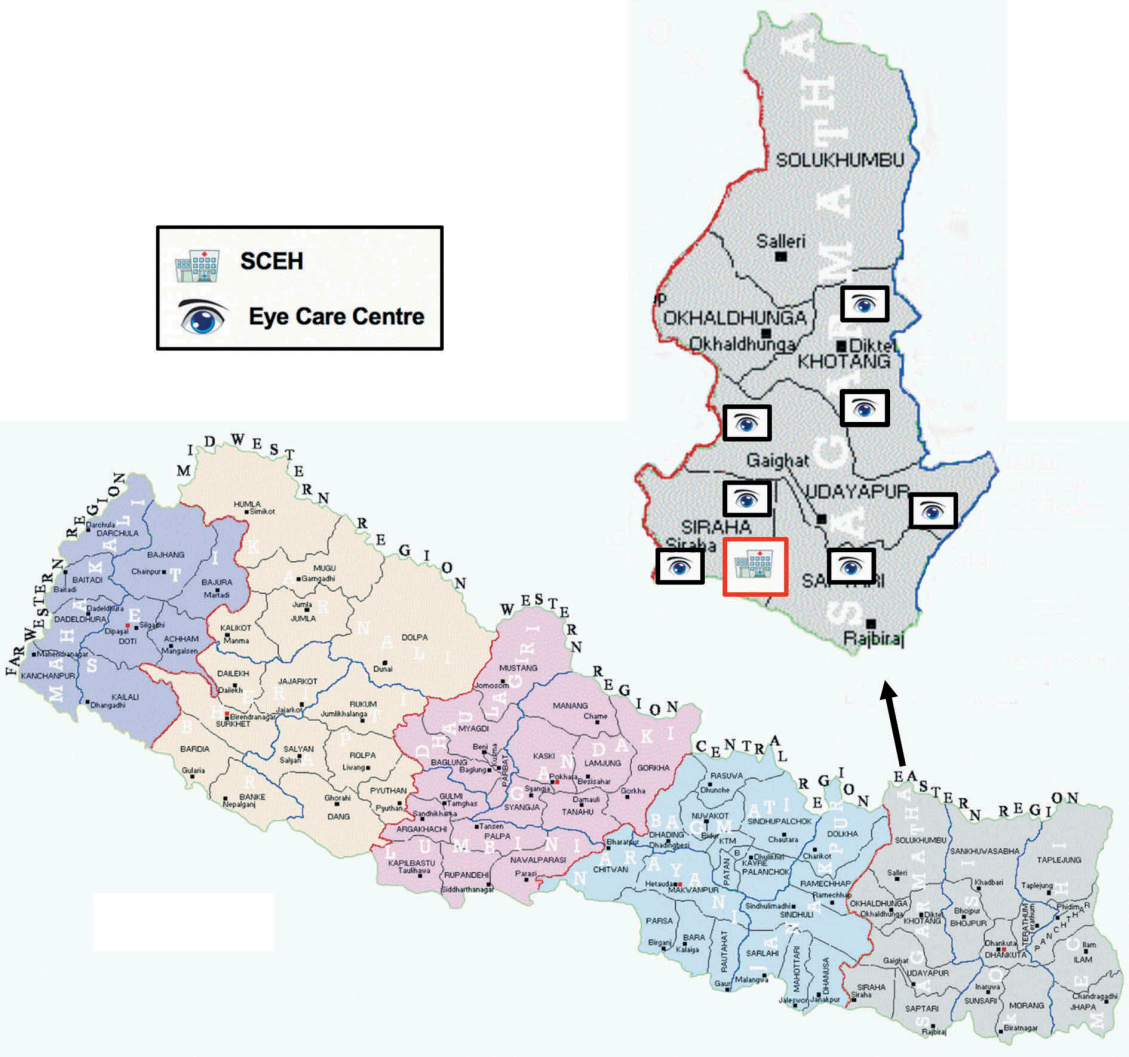
Map of eye care provision in Sagarmatha zone, Nepal.

Nepal provides an important example of a well-established eye care system functioning within a low-income country. Since the first national blindness prevalence survey in 1981 eye care provision in Nepal has expanded greatly and the prevalence of blindness has significantly reduced.^17^ There are 35 secondary or tertiary eye hospitals, 96 community eye care centres (ECCs) and 254 ophthalmologists.^18^ The ECCs are staffed by ophthalmic assistants, optometrists and eye health workers and provide diagnosis, treatment, rehabilitation and referral of eye conditions. There is approximately one per district, however eye care provision does not reach beyond the district level of health care provision into the community. Nepal is divided into three geographical regions along a south to north transect; the terai plains (bordering India to the south), the hills and the mountains (bordering Tibet to the north). Most eye hospitals are in the terai, and most ECCs do not reach into the mountain regions. Sagarmatha zone (Everest zone) in Eastern Nepal has a population of approx. 2.06 million people with 1 tertiary eye hospital and 7 community eye care centres (Figure 1).^19^ Similar to Nepal as a whole, cataract is the main cause of blindness accounting for 66.7%, followed by posterior segment disease (10.3%) and non-trachomatous corneal opacity (5.1%).^20^ Most of the eye services are run by the NGO sector with little integration with the government run health service.

The Nepal Vision 2020 mid-term review states integration with primary health care is vital for reducing blindness in Nepal.^17^ However, we do not know what level of PEC provision by PHCWs is currently provided and what their role should be within an integrated service. Studies elsewhere have consistently shown that knowledge and skills of PEC is limited, but no such study has been carried out in Nepal. This study aims to assess the current practise of PHCWs in eye care including the infrastructure and resources available, their attitudes towards PEC and their level of knowledge, skills and training.

## Materials and methods

A mixed methods descriptive cross-sectional study with semi-structured interviews and focus group discussions was carried out in Sagarmatha zone, Eastern Nepal between 13^th^ June and 22^nd^ July 2018.

Approval was granted by the London School of Hygiene and Tropical Medicine Ethics Committee and Sagarmatha Choudhary Eye Hospital Lahan (SCEH) Institutional Management Board. All participants were provided with an information sheet in Nepali. Written informed consent was provided by each participant. All data collected on paper questionnaires were anonymised and interviews were recorded on an encrypted smartphone. This study was carried out according to the principles of the Declaration of Helsinki.

### Quantitative methodology

#### Study location and population

The study population for the quantitative study were PHCWs working in Sagarmatha zone, Nepal. These included doctors working in the community, nurses, health assistants (HAs), auxiliary health workers (AHWs), community medical assistants (CMAs) and auxiliary nurse midwives (ANMs). The inclusion criteria were (i) PHCWs employed by the Nepali government, (ii) consent to take part, (iii) currently manage eye patients in their health facility.

#### Sample size and strategy

The sample size was calculated for a precision of 10%, confidence interval of 95% and an expected proportion of 50% of PHCWs being able to accurately diagnose cataract from a clinical photograph. A design effect of 1.05 was added to account for clustering within the health care facilities. A sample size of 103 PHCWs was required. Assuming an average of 3 PHCWs in each health post, a total of 35 health posts were required.

Siraha, Udayapur and Khotang districts of Sagarmatha zone were purposely selected to represent the terai, hills and mountain regions, respectively. Within each district individual health posts (HPs) were selected. HPs that were not accessible by road or within one day’s drive were excluded. 15 HPs in Siraha and 15 Udayapur were selected via simple random sampling using a random number generator. In Khotang, due to difficult roads during the monsoon season only 6 were selected.

#### Data collection

**Figure 2.**
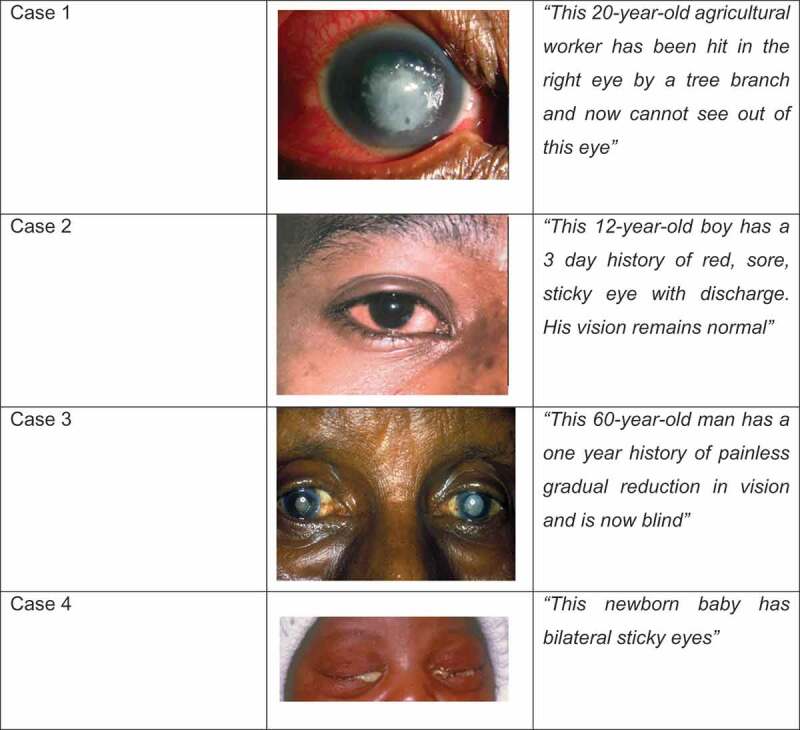
Clinical cases used in knowledge assessment.

During the study period HPs were visited by the primary researcher and local guide/translator. All government health workers present at the time of the visit were invited to take part. The questionnaire was administered by the primary researcher with translation into Nepali. Part 1 asked demographic and training information and part 2 tested PEC knowledge and skills. Participants were asked whether they had received any eye care training since starting their professional career as a PHCW, post primary qualification. Each participant was shown colour clinical images alongside a brief clinical scenario for four common eye conditions and asked if they could diagnose the condition (1 point), manage the condition (1 point) and how urgent they felt the condition was (1 point) (Figure 2). They were also asked whether they would refer the patient and if so where would they refer to. Participants were then asked if they had been trained to test visual acuity. If they had been, 1 point was awarded for each of the following: measuring visual acuity from 6 metres, measuring each eye separately, reading the correct visual acuity from vision chart, correctly interpreting the visual acuity result. After each interview the translator explained any incorrect answers to the participant to provide one-to-one eye health education. Each health post was assessed for a distance vision testing chart, near vision testing chart, working torch, magnifying loupe, pin hole occluder and any eye medications currently in stock.

#### Quantitative data analysis

Data was cleaned and exported to Stata v.15 for analysis. A statistical test of difference in total scores for the diagnosis, management and timing of management for each case was carried out using χ^2^ analysis. The following explanatory factors were tested for an association with total skill score: previous eye training, sex, professional cadre, district. An independent t-test was used for continuous variables with binary categories and one-way ANOVA was used for those with more than two categories. Logistic regression was used to calculate the odds ratio and 95% confidence interval of providing the correct diagnosis for those who had received previous eye training. Where tabulated values were 0 for any category, exact logistic regression was used.

### Qualitative methodology

#### Participants and sampling

Three focus group discussions took place, one in each of the included districts. Five PHCWs from each district who had taken part in the quantitative study were purposely selected for each FGD. Participants were chosen to provide a range of cadres, geographic locations and sex. All 7 ophthalmic assistants (OAs) working in the ECCs in the 3 selected districts and the district health manger were invited to take part in semi-structured interviews.

#### Data collection

The FGD was conducted in Nepali with real-time translation into English. The discussion was recorded, transcribed and translated into English by the translator. During the interview questions were asked to explore the attitudes of PHCWs towards the delivery of eye care, its challenges and how services could be improved.

The semi-structured interviews were carried out by the principal researcher in English with real-time translation into Nepali. Ophthalmic assistants were asked about their relationship with the health posts, the feasibility of integration with PHCWs and how community ophthalmic services can be improved. The interview with the district health manager explored the role of government in providing eye care and current community eye care provision. The interviews were recorded and transcribed in English by the principal researcher.

#### Qualitative data analysis

The transcripts were read through several times for a process of familiarisation and reflection. A coding system was then developed using an iterative process of code development. Initial codes were developed from analysis of the first few transcripts and then expanded. The codes were then grouped into themes from which key quotes were identified to illustrate these themes. Triangulation was carried out to compare and contrast the views of the OAs, PHCWs and the district health manager, and the qualitative with the quantitative data. Analysis was continued until saturation was reached; whereby no additional data led to new emergent themes.

## Results

### Participants

**Figure 3.**
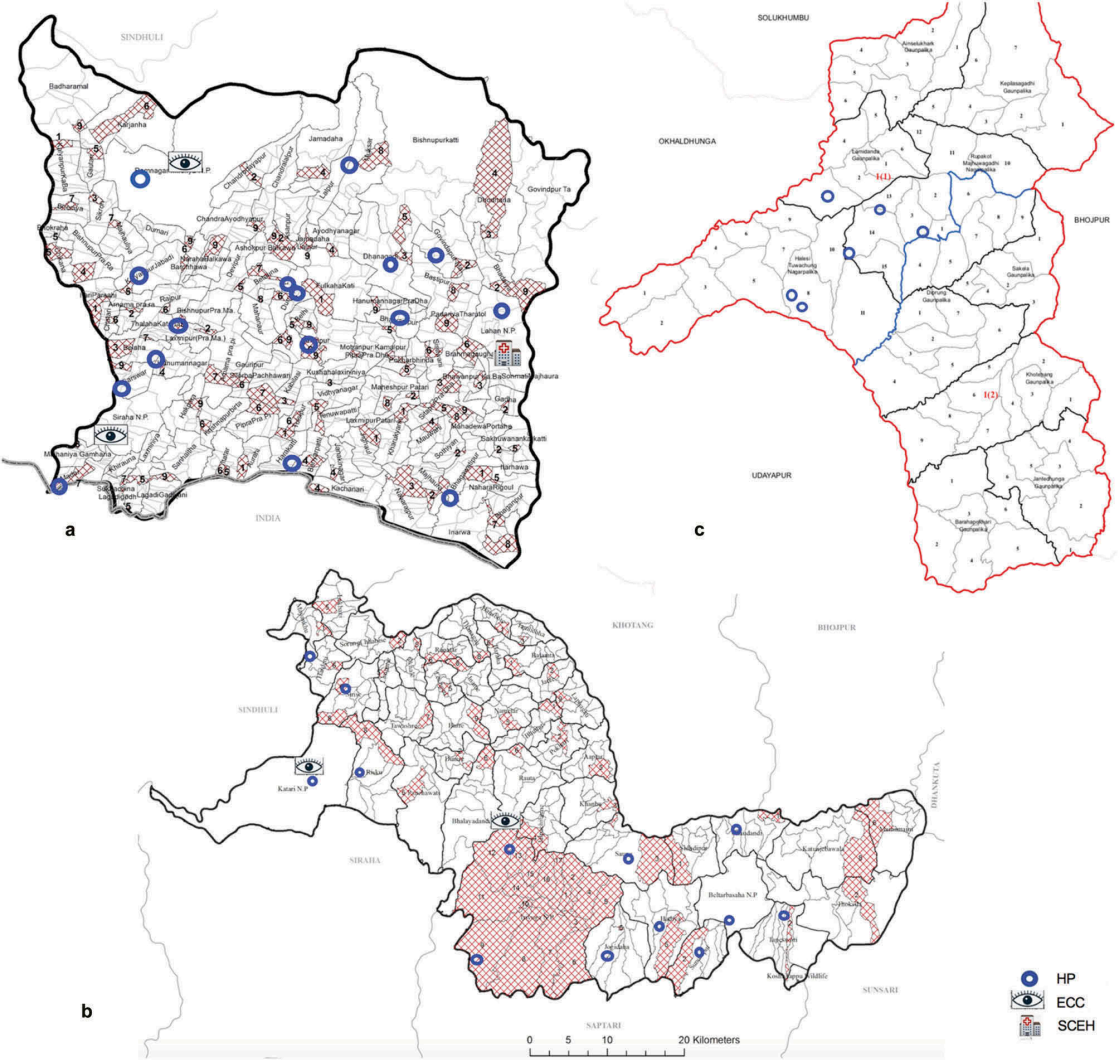
Map of health posts visited in each district A. Siraha B. Udayapur C. Khotang.

A total of 107 PHCWs from 35 health posts (HP) participated in the quantitative study; 16 HPs in the terai (46 participants), 13 HPs in the hills (40 participants) and 6 HPs in the mountains (21 participants) (Figure 3) (Table 1). All staff present at each HP took part (100% response rate). The most common cadre of staff that participated was auxiliary nurse midwives (ANMs, 39.3%). The majority of participants were female (54.2%) and their mean age was 35 years. Approximately half of all participants had been working for 5 years or less. Table 2 describes the participants of the qualitative study. We conducted semi-structured interviews with seven OAs and one district health manager. We conducted three FGDs, each included five participants.

**Table 1.** Demographic and training characteristics of participants in the quantitative part of the study.

Location	Terai	Hills	Mountains	Total
Participants	n/46 (%)	n/40 (%)	n/21 (%)	n/107 (%)
Age				
20–24	7 (15.2)	5 (12.5)	2 (9.5)	14 (13.0)
25–29	7 (15.2)	3 (7.5)	11 (52.4)	21 (19.6)
30–34	6 (13.0)	11 (27.5)	4 (19.0)	21 (19.6)
35–39	8 (17.4)	9 (22.5)	2 (9.5)	19 (17.8)
40-44	7 (15.2)	4 (10.0)	0 (0.0)	11 (10.3)
45-50	5 (10.9)	4 (10.0)	2 (9.5)	11 (10.3)
>50	6 (13.0)	4 (10.0)	0 (0.0)	10 (9.3)
Mean (sd)	36.26 (1.5)	36.1 (1.4)	30.14 (1.5)	35.00 (0.9)
Sex				
Male	30 (65.2)	12 (30.0)	7 (33.3)	49 (45.8)
Female	16 (34.8)	28 (70.0)	14 (66.7)	58 (54.2)
Staff by cadre				
Nurse	4 (8.7)	1 (2.5)	1 (4.8)	6 (5.6)
CMA	11 (23.9)	6 (15.0)	5 (23.8)	22 (20.6)
HA	10 (21.7)	6 (15.0)	3 (14.3)	19 (17.8)
ANM	10 (21.7)	23 (57.5)	9 (42.9)	42 (39.3)
AHW	10 (21.7)	3 (7.5)	3 (14.3)	16 (14.9)
Doctor	1 (2.2)	0 (0.0)	0 (0.0)	1 (0.9)
Lab Assistant	0 (0.0)	1 (2.5)	0 (0.0)	1 (0.9)
Duration of training				
< 1 year	8 (17.4)	3 (7.5)	1 (4.8)	12 (11.2)
15 months	7 (15.2)	5 (12.5)	8 (38.1)	20 (18.7)
18 months	11 (23.9)	24 (60.0)	8 (38.1)	43 (40.2)
2 years	8 (17.4)	1 (2.5)	0 (0.0)	9 (8.4)
3 years	10 (21.7)	7 (17.5)	4 (19.1)	21 (19.6)
>3 years	2 (4.3)	0 (0.0)	0 (0.0)	2 (1.9)
Years of work				
< 1	6 (13.0)	12 (30.0)	5 (23.8)	23 (21.5)
1–4	13 (30.4)	9 (22.5)	11 (52.4)	34 (31.8)
5–9	7 (15.2)	4 (10.0)	0 (0.0)	11 (10.3)
10–14	3 (6.5)	2 (5.0)	2 (9.5)	7 (6.5)
15–19	3 (6.5)	8 (20.0)	2 (9.5)	13 (12.1)
>20	13 (28.3)	5 (12.5)	1 (4.8)	19 (17.8)
Provide eye care	46 (100.0)	40 (100.0)	21 (100.0)	107 (100.0)
Eye care training as a professional	6 (13.0)	2 (5.0)	1 (4.8)	9 (8.4)

**Table 2.** Participants for semi-structured interviews and FGDs.

Location	Cadre	Sex
***Semi-structured interview***		
Terai	OA	M
	OA	M
	OA	F
Hills	OA	M
	OA	M
Mountains	OA	M
	OA	M
Regional	DHM	M
***Focus Group Discussions***		
Terai	HA	M
	HA	M
	HA	M
	ANM	F
	CMA	M
Hills	HA	M
	HA	M
	CMA	M
	ANM	F
	ANM	F
Mountains	HA	M
	HA	M
	CMA	M
	CMA	F
	ANM	F

OA Ophthalmic Assistant; DHM District health manager; HA health assistants; ANM auxiliary nurse midwives; CMA community medical assistants

### Knowledge and skills

The results of the clinical skills test are shown in Table 3. In all three districts we found that corneal ulcer was the condition with the lowest recognition score (27%) and only 58.8% could explain the correct management. Similarly, the scores for the diagnosis and management of ophthalmia neonatorum were also poor. The recognition rates for both cataract and conjunctivitis were somewhat better. Only 14% of participants had been trained to measure visual acuity (Table 3). Encouragingly, among those that had been trained, many were still able to measure visual acuity.

**Table 3.** Clinical skills test. The number of PHCWs with provided the correct answer for the diagnosis, management and timing (urgency) of four clinical conditions.

	Total N/107 (%)	Terai n/46 (%)	Hills n/40 (%)	Mountains n/21 (%)	p-value
**Corneal ulcer**					
Diagnosis	29 (27.1)	18 (39.1)	8 (20.0)	3 (14.4)	0.05
Management	63 (58.9)	26 (56.5)	26 (65.0)	11 (52.4)	0.58
Timing	74 (69.2)	28 (60.9)	33 (82.5)	13 (61.9)	0.07
Median score (IQR)	2 (1–2)	2 (0–3)	2 (1–2)	1 (0–2)	
Mean score (SD)	1.55 (0.10)	1.57 (0.17)	1.68 (0.15)	1.29 (0.24)	
**Conjunctivitis**					
Diagnosis	89 (83.2)	42 (91.3)	31 (77.5)	16 (76.2)	0.15
Management	94 (87.9)	42 (91.3)	35 (87.5)	17 (81.0)	0.48
Timing	71 (66.4)	30 (65.2)	28 (70.0)	13 (61.9)	0.80
Median score (IQR)	3 (2–3)	3 (2–3)	3 (2–3)	3 (2–3)	
Mean score (SD)	2.37 (0.09)	2.48 (0.12)	2.35 (0.15)	2.19 (0.26)	
**Cataract**					
Diagnosis	81 (75.7)	37 (80.4)	29 (72.5)	15 (71.4)	0.61
Management	74 (69.2)	34 (73.9)	26 (65.0)	14 (66.7)	0.65
Timing	45 (42.0)	28 (60.9)	12 (30.0)	5 (23.8)	0.01
Median score (IQR)	2 (1–3)	3 (2–3)	2 (0.5–2.5)	2 (0–2)	
Mean score (SD)	1.87 (0.11)	2.15 (0.16)	1.68 (0.18)	1.62 (0.25)	
**Ophthalmia neonatorum**					
Diagnosis	58 (54.2)	29 (63.0)	20 (50.0)	9 (42.9)	0.24
Management	56 (52.3)	24 (52.2)	20 (50.0)	12 (57.1)	0.87
Timing	66 (61.7)	27 (58.7)	28 (70.0)	11 (52.4)	0.35
Median score (IQR)	2 (1–3)	2 (1–3)	2 (1–3)	2 (1–2)	
Mean score (SD)	1.68 (0.10)	1.74 (0.16)	1.70 (0.16)	1.52 (0.21)	
**Total Score**					
Median score (IQR)	8 (6–9)	8 (6–10)	7.5 (6–9)	7 (6–8)	
Mean score (SD)	7.48 (2.46)	7.93 (2.29)	7.40 (2.41)	6.62 (2.77)	0.60*
**Visual acuity training**					
Yes	15 (14.0)	9 (19.6)	4 (10.0)	2 (9.5)	0.36
No	92 (85.9)	37 (80.4)	36 (90.0)	19 (90.5)	
Median VA score (IQR)	3 (2–4)	3 (3–4)	2.5 (0.5–4)	3.5 (3–4)	
Mean VA score (SD)	2.88 (0.35)	3.00 (0.44)	2.25 (1.03)	3.33 (0.33)	

The maximum score for each condition was 3 and the maximum total score was 12. The maximum visual acuity score was 4; this only included those that had been trained. The *p*-value is for a comparison of the three districts. *p-value calculated by ANOVA.

Only four participants out of 107 achieved the maximum score of 12 in the clinical skills test. Overall, PHCWs in the terai had the highest scores, followed by the hills and then the mountains. There was no difference in mean total scores by sex (Table 4). There was no difference between men and women receiving previous eye care training (*p* = 0.53). The differences in scores between professional cadres were not statistically significant; ANMs had the lowest mean score (6.62) while accounting for almost half of the staff available at health posts managing eye patients.

**Table 4.** Clinical skills test. Potential explanatory factors, associated with total overall knowledge score (out of a maximum of 12).

	Mean Total	(S.D.)	p-value
***Previous eye training***			
Yes	9.22	(2.41)	0.03*
No	7.32	(2.49)	
***Sex***			
Male	7.96	(2.67)	0.06*
Female	7.06	(2.39)	
***Years worked***			
<5 years	7.41	(2.58)	0.75*
>5 years	7.57	(2.32)	
***Professional cadre***			
Nurse	8.00	(2.37)	0.79**
HA	8.63	(2.27)	
CMA	7.45	(2.70)	
AHW	7.18	(1.97)	
ANM	6.62	(2.36)	
***District***			
Terai	7.93	(2.29)	0.60**
Hills	7.40	(2.41)	
Mountains	6.62	(2.77)	

*calculated using independent t-test; **calculated using one-way ANOVA. OA Ophthalmic Assistant; DHM District health manager; HA health assistants; ANM auxiliary nurse midwives; CMA community medical assistants.

Interviews with OAs revealed concerns about the low level of PEC knowledge among PHCWs in all three regions, in particular the incorrect management of corneal ulcers: *“I don’t think they have any knowledge about the eye, if corneal ulcer found they use steroid which is very bad … steroid is completely to be avoided, but they don’t know”* [OA hills].

PHCWs stated that they can manage simple conditions such as conjunctivitis, but they do not have adequate knowledge of other ophthalmic conditions: *“we receive many kinds of eye problems but not many ideas of how to manage them … we lack knowledge and proper skill. I just provide primary care, just prescribing Ciprofloxacin eye drop.”* (PHCW terai).

Only 69.2% of participants correctly recognised corneal ulcer as an emergency and 54% recognised ophthalmia neonatorum as an emergency requiring immediate treatment and referral. This is supported by the qualitative data: *“they [PHCWs] give medicine which is not correct and wait 7 to 10 days. After the problem is not solved then they send to us [OA]. It’s too late and it causes too much problem”* [OA mountains].

### Equipment and medication

We visited 35 health posts to assess the availability of equipment and ophthalmic medication (Table 5). Only 2 (12%) HPs had a distance vision chart. Working torches were found in about two thirds of HPs in the terai and hills, but only in one HP in the mountains. Nearly all HPs had stocks of ciprofloxacin eye drops. None of the centres had fluorescein or other ophthalmic medications. These direct observations were consistent with the qualitative reports:

“… no adequate resources, logistics and equipment in the health post for delivering eye care services … it is not impossible for us to check vision but due to lack of resources and equipment it is difficult. Basic equipment such as torch government has not provided.” [PHCW terai].

**Table 5.** Equipment, ophthalmic medication and eye patient presentations at health posts.

	Siraha n/16 (%)	Udayapur n/13 (%)	Khotang n/6 (%)
Distant vision chart	2 (12.5)	0 (0.0)	0 (0.0)
Near vision chart	0 (0.0)	0 (0.0)	0 (0.0)
Working torch	11 (68.6)	8 (61.5)	1 (16.7)
Loupe	0 (0.0)	0 (0.0)	0 (0.0)
Ophthalmic antibiotics	15 (93.8)	13 (100.0)	6 (100.0)
Ciprofloxacin eye drops	14 (87.5)	13 (100.0)	6 (100.0)
Tetracycline eye drops/ointment	4 (25.0)	0 (0.0)	0 (0.0)
Chloramphenicol eye drops/ointment	5 (31.2)	1 (7.7)	0 (0.0)
Fluorescein	0 (0.0)	0 (0.0)	0 (0.0)
Other ophthalmic medications	0 (0.0)	0 (0.0)	0 (0.0)
Mean no. of eye patients/month/health centre	12.98	15.10	6.39

### PEC training

The only PHCWs that have eye care included in their pre-qualification training curriculum are doctors, nurses, CMAs and HA’s. Only 8.4% of PHCWs had received eye care training during their professional career post qualification. (Table 2). Those that had received eye training as a professional performed slightly better on the knowledge and skills questionnaire compared to those that had not received training (Table 4). The association between having received some professional PEC training and the making of a correct diagnosis is shown in Table 6. There appear to be non-significant trends in favour of making a correct diagnosis if the individual had received some professional PEC training.

**Table 6.** Primary eye training and making a correct diagnosis, analysed by univariate logistic regression.

	PEC Trained		No PEC Training				
	n/9	(%)	n/98	(%)	OR	95% CI	p-value
Corneal ulcer	4	(44%)	25	(26%)	2.34	0.58–9.39	0.23
Conjunctivitis*	9	(100%)	80	(82%)	2.72	0.40 – ∞	0.35
Cataract*	9	(100%)	72	(73%)	4.38	0.65 – ∞	0.14
Ophthalmia neonatorum	6	(67%)	52	(53%)	1.77	0.42–7.48	0.44

* Exact logistic regression used.

The OAs, PHCWs and district health manager all agree that PEC training is required in order to reduce the burden of blindness in their communities and all PHCWs stated they would be willing to receive training and that they had the time to take on this additional role.

*“Very much necessary to train the government staff in the eye care system … to provide some knowledge, eye awareness programme so that they know how complicated the eye is.”* [OA mountains].

“If we receive some training we can provide eye health education to our patients, better treatment and early referral” [PHCW terai].

### Referral pathways and communication

Patients with eye conditions were seen several times a week at most of the HPs (Table 5). The conditions most likely to be referred by all three districts are corneal ulcers (78.9% of PHCWs would refer) and cataract (78.9%), followed by ophthalmia neonatorum (78.2%); few would refer conjunctivitis (27.6%). In the terai on average across all four conditions 94.3% of PHCWs would refer to SCEH. In contrast, only 20.7% of PHCWs working in the mountains would refer patients to SCEH, most would refer only to the local ECC. All OAs and PHCWs stated it was important in the elimination of blindness to have good communication and collaboration between the two services. However, none stated that they had yet established this close working relationship.

*“it is important that we all work together to solve this problem of blindness … but we have no direct contact with eye hospital. No phone calls, no communication. We feel we are doing as much as we can but we do not receive feedback.”* [PHCW terai].

### Patient barriers

The distance between HPs and ECCs, lack of patient awareness of eye diseases and cost were three patient barriers to receiving eye care consistently mentioned by OAs and PHCWs working in the hills and mountains.

“patients have to walk too much long time 3,4,6 hours to come from their home to eye care centre it is very far. We are not able to reach into community properly” [OA mountains].

“health seeking behaviour is still very poor. People are not getting treatment early enough … not just us at the health post level but communities need to be sensitised about the eye.” [PHCW hills].

### The role of government

OAs and PHCWs described the need for greater government commitment and support for eye care in order to improve eye care services and reduce the prevalence of blindness and visual impairment. *“The Nepal government does not give any type of support to the eye care centre, they don’t do any sort of eye health activity at the village level or eye care centre or even hospitals. The Nepal government is not undertaking the eye health system as much as other health. I have received many trainings over the years in TB, family planning, leprosy but never in eyes”*. [PHCW terai].

## Discussion

This is the first study to assess the knowledge, skills and attitudes of PHCWs towards PEC in Nepal. A range of PHCWs participated with differing primary clinical responsibilities, lengths of training and training curriculum content. While all PHCWs stated that they provide eye care to patients, very few had received eye care training during their professional career, and many had no eye care component in their training curriculum for qualification. Having received eye care training during their professional career was associated with achieving a slightly higher PEC knowledge and skill score. This finding highlights an important gap in eye care training within this region. It is consistent with the views expressed by PHCWs, OAs and the district health manager, who all agreed that specific PEC training would improve eye care provision. All PHCWs were keen to receive such training, felt that primary eye care provision was part of their role and stated that they had the time to gain these extra skills. The OAs emphasised that patients currently come to harm from the lack of knowledge and skills of the PHCWs and that training would be the best way to improve this.

Overall, the PEC knowledge and skills scores were low. Higher scores were associated with having professional eye care training, but not with gender, professional cadre or geographical location. Those working in the mountain regions, and therefore furthest away from the eye hospital, performed less well for all four eye conditions. The most concerning finding was the very low number of PHCWs who could accurately diagnose, manage and make an emergency referral for corneal ulcer and ophthalmia neonatorum. Knowledge of corneal ulcers was particularly poor in the mountain region, where the travel time to the closest eye care centres is very considerable and therefore the need for good PEC in the community is greater. Nepal has one of the highest incidences of corneal ulcers worldwide (799/100,000/year)^21^ and non-trachomatous corneal opacity is the third leading cause of blindness in this region.^20^ As such it is of great importance that this condition can be reliably recognised and patients receive early primary treatment and prompt referral. The Bhaktapur eye study showed that the progression of a corneal abrasion to an infected corneal ulcer could be reduced to almost zero by the use of prophylactic antibiotics by PHCWs.^21^ No HP visited had a blue torch, fluorescein eye drops or training on how to carry out this simple examination.

Cataract was correctly recognised by 75% of PHCWs. This is higher when compared to other similar studies: 56% in Tanzania, Malawi and Kenya, 49.5% in Ethiopia and 72.3% in Nigeria.^12–14^ This number is still low, however, considering cataract remains the leading cause of blindness in the Sagarmatha region despite surgical provision by SCEH and suggests that recognition at the community level needs to be improved in order to reduce cataract blindness.

The number of PHCWs who had been trained to measure visual acuity was very low (14%) and the number of HPs equipped with a distance vision chart was even lower (5.7%). This basic skill is an important component of PEC and is largely missing from PEC provision in this region. In addition, HPs had very little other ophthalmic equipment, including even basic tools such as a working torch. The PHCWs explained they were keen to provide eye care but did not have the resources and equipment to be able to do so.

We found that HPs are a frequent contact point for people with eye problems, and function as referral sources to move eye patients through the health care system to access specialised eye care. For all conditions, except conjunctivitis, 70–80% of PHCWs would refer patients to another centre. It appears that the ECCs are used far more as a referral facility in the hills and mountains than in the terai, where patients are referred directly to SCEH. This suggests that the community eye services may benefit from an increase in these services in the hills and mountains and fewer in the terai, although these findings would need to be corroborated by the eye patient perspective.

The views of PHCWs about the role of the government health sector in providing eye care and the integration of eye care into primary health care was in line with that outlined by the Vision2020 mid-term review. The eye care provision in Nepal runs as a vertical system, mostly provided by local NGO’s rather than government, and has achieved great reductions in the prevalence of blindness since its establishment in the 1980s. This separation between the Nepal government health sector and the organisations providing eye care is likely to be contributing to the low level of eye care knowledge and practice among primary health care workers, as they are trained and work within the government system. Greater integration of eye care provision within the government health sector may help to increase the quantity and quality of eye care training for primary health care workers.

### Limitations

The first limitation of the study is that unequal numbers of HPs and participants were sampled from the three districts due to difficult access during the monsoon season. In particular this led to under representation of the mountain region in the quantitative study which may introduce selection bias. Secondly, the study was not statistically powered to provide a comparison between the three regions because there were not large enough sample sizes in all three regions. Where statistically significant differences between the regions are not shown to exist we must be aware that there may in fact be a real difference, but the study was under powered to detect this difference. Thirdly, there are several potential biases in the qualitative data. Researcher bias is an important bias which occurs through the interviewer having their own beliefs, preconceptions and personal experiences which may influence how questions are asked, and how answers are analysed.^22^ We aimed to provide a neutral position while conducting and analysing the interviews, however the role of the researcher must always be considered when interpreting this data. As purposive sampling was carried out for the qualitative component there is potential for selection bias as those participating in the study may not be representative of all health workers in the region. In particular in interviewing one district manager we did not capture a range of views from those in managerial positions. Lastly, the quality of data collected in the qualitative interviews may have been affected by the language barrier between the principal researcher and the subject leading to questions becoming more closed or the interpreter altering the meaning of the subject’s response.

### Conclusion

Vision 2020 and the Global Action Plan 2013–2019 both prioritise PEC as a key component to improving the uptake of ophthalmic services and reducing avoidable blindness. This is the first study in Nepal to investigate the level of PEC knowledge and skills of those working at the primary care level. We found that they are currently inadequate to provide the basic recognition, management and referral expected from the broad definition of PEC. Eye care specialists managing eye patients in the community are concerned that the current provision of PEC is inadequate, likely due to poor provision of eye care training. Those working in primary health care have a desire for PEC training and ophthalmic equipment and view themselves as a key component of the fight to end avoidable blindness in their community. This study highlights that to improve PEC there needs to be greater government support for ophthalmic training and resource provision, and advocacy for the integration of PEC with existing primary health care.

## Submission Declaration

This submission has not been published anywhere previously and it is not simultaneously being considered for any other publication

## Appendix 1. Focus Group Discussions: Interviewer Guide

1. **Do you feel blindness and eye diseases are a problem in your district?**
  - If yes - why do you think eye diseases are a problem in your district
2. **What do you feel is the role of Health Posts with regards to providing eye care?**
  - Is providing eye care the responsibility of the health post?
  - Is it only the responsibility of the eye care centre?
3. **How well do you think your health post is able to manage eye patients?**
  - Do you encounter problems in providing eye care?
  - If so, what are these problems?
    - Resources and equipment?
    - Lack of training?
4. **Do you think changes could be made to improve treatment of eye patients in your health post?**
  - Would you like to have training in how to recognise, treat and refer common eye conditions?
5. **Do you feel you can take on this additional training and role?**
  - Do you have enough time?
  - Is eye care a priority?
6. **To what extent do you feel connected to the Eye Care Centres in your district?**
  - Are you aware the eye care centres are there?
  - Do you refer patients to the eye care centre?
  - Do you ever receive feedback from them?
7. **To what extent do you feel supported by the Eye Care Centre in your district?**
  - What do you think this eye care service could do to help you manage eye patients better in your health posts?

**Semi-Structured Interviews with Ophthalmic Assistants: Interviewer Guide**

1. Please describe to me how your eye care centre is linked to health posts near by
  - Do you have a referral system between the two health centres?
  - Do you have communication with the health posts regarding eye patients?
2. Can you describe how eye patients in your district access eye care?
  - Do you see many patients that have first attended the health post?
  - Do patients come straight to the eye care centre?
3. How well do you feel primary health workers in health posts are able to manage eye patients?
4. Do you think training primary health workers to recognise and refer important eye problems to the primary eye care centre would be helpful?
5. What do you think would be the most useful skills and knowledge for primary health workers to have regarding eye care?
6. Who do you think would be best placed to carry out eye care training for primary health workers?
  - Ophthalmic assistants?
  - Trainers at Lahan?
7. How do you think communication between the health post and eye care centre can be improved?
8. In general, what do you think could be done to improve eye care services at the community level in your district?
